# Efficacy and Safety of 12-week Monotherapy With Once Daily 5 mg Tadalafil for Lower Urinary Tract Symptoms of Benign Prostatic Hyperplasia: Evidence-based Analysis

**DOI:** 10.3389/fmed.2021.744012

**Published:** 2021-10-12

**Authors:** Jianwei Cui, Dehong Cao, Yunjin Bai, Jiahao Wang, Shan Yin, Wuran Wei, Yunfei Xiao, Jia Wang, Qiang Wei

**Affiliations:** Department of Urology, Institute of Urology, West China Hospital of Sichuan University, Chengdu, China

**Keywords:** benign prostatic hyperplasia (BPH), lower urinary tract symptoms (LUTS), tadalafil, efficacy, safety

## Abstract

**Background:** Tadalafil has been approved for the treatment of benign prostatic hyperplasia (BPH) for nearly 10 years. However, there are insufficient evidence-based studies of the efficacy and safety of tadalafil in treating lower urinary tract symptoms of BPH (LUTS/BPH).

**Objective:** To evaluate the therapeutic effect and clinical safety of tadalafil monotherapy (5 mg once daily for 12 weeks) for LUTS/BPH.

**Methods:** A total of 13 studies (15 randomized clinical trials [RCTs]) were extracted from the following databases: PubMed, Cochrane Central Register of Controlled Trials, Embase, and Web of Science for the period up to July 2021. The quality of the included RCTs was evaluated independently by two authors, who, respectively, extracted data according to the Preferred Reporting Items for Systematic Reviews and Meta-Analyses principles. Conflicts were settled by a discussion with two-third of senior authors. All data analyses were conducted by the Review Manager, version 5.4.

**Results:** Regarding efficacy, 12-week trials indicated that 5 mg once daily tadalafil showed a significantly lower and, consequently, better total International Prostate Symptom Score (IPSS) than the placebo did (mean difference [MD]: −1.97, 95% CI: −2.24 to −1.70; *P* < 0.00001). In addition, significant differences were found between the tadalafil regimen and the placebo in the IPSS voiding subscore (MD: −1.30, 95% CI: −1.48 to −1.11; *P* < 0.00001), the IPSS storage subscore (MD: −0.70, 95% CI: −0.82 to −0.58; *P* < 0.00001), the IPSS quality of life (MD: −0.29, 95% CI: −0.35 to −0.22; *P* < 0.00001), and BPH impact index (MD: −0.58, 95% CI: −0.76 to −0.40; *P* < 0.00001). The safety analysis did not show a significant difference in serious adverse events between the two groups (risk ratio: 1.27, 95% CI: 0.80–2.01; *P* = 0.31), although the adverse events occurred at a higher incidence in the tadalafil group than in the placebo.

**Conclusions:** This study demonstrates that once daily 5 mg tadalafil is a potentially effective and safe treatment choice with excellent tolerability for patients with LUTS/BPH.

**Systematic Review Registration:** Identifier (CRD42021228840).

## Introduction

Benign prostatic hyperplasia (BPH), which is caused by the proliferation of epithelial and stromal cells in the transition zone of the prostate, is characteristic of nonmalignant hyperplasia of prostatic tissue and is highly prevalent in older men (1). Lower urinary tract symptoms (LUTS) associated with BPH include storage or irritative (mainly including urinary frequency, urgency, and nocturia), voiding or obstructive (mainly including urinary hesitancy, straining, retention, and a decreased force of urination), and postmicturition symptoms, which can significantly and negatively affect the quality of life (QoL) of the elderly (2, 3). More than 50% of men > 50 years and over 80% of men > 80 years old experience LUTS/BPH (4).

In addition to surgical intervention, alpha-blockers (ABs), 5-alpha reductase inhibitors (5ARIs), and phytotherapies (monotherapy or cotherapy) have been prescribed for the treatment of LUTS related to BPH for decades (5, 6). Although these drug treatments are effective, they can cause troublesome adverse reactions, such as hypotension, dizziness, and sexual dysfunction (7, 8). Tadalafil, one of the phosphodiesterase type 5 inhibitors (PDE5Is) used to improve the symptoms of erectile dysfunction (ED), has been increasingly investigated and gained approval for the treatment of BPH in many countries since 2011 (9).

This is mainly because PDE5Is are believed to likely act mainly by increasing the concentration of nitric oxide (NO), which improves the activity of intracellular cyclic guanosine monophosphate (cGMP) (10). This system is collectively called the NO-cGMP signaling pathway, and its activation could lead to the relaxation of smooth muscle in the detrusor, bladder neck, and prostate (10). In addition, PDE5Is not only ameliorate intraprostatic inflammation-associated BPH but also improve tissue oxygenation and blood supply and thereby playing a vital part in alleviating LUTS (9, 11).

Recent meta-analyses comparing the effect of tamsulosin as a monotherapy and a cotherapy with tadalafil in improving LUTS/BPH, have concluded that cotherapy might be more suitable for patients than monotherapy (12, 13). However, analyses of tadalafil 5 mg monotherapy administered once daily for the treatment of LUTS/BPH are rare. Despite the use of tadalafil monotherapy, LUTS/BPH has been reported to improve significantly following treatment with this strategy (14). Therefore, in this study, we conducted the latest and high-quality synthesis of current worldwide evidence to evaluate the therapeutic effect and clinical safety of tadalafil 5 mg once daily monotherapy for 12 weeks in men with LUTS/BPH.

## Materials and Methods

### Study Protocol

The study registration application has been submitted to the International Prospective Register of Systematic Reviews (CRD42021228840) and the report was prepared using the Preferred Reporting Items for Systematic Reviews and Meta-Analyses guidelines.

### Information Sources and Literature Search

A comprehensive and systematic search of PubMed, Cochrane Central Register of Controlled Trials, Embase, and Web of Science databases was performed for the period up to July 2021 by using the following key terms: [(“Prostatic Hyperplasia”[Mesh]) OR (Benign Prostatic Hyperplasia) OR (Hyperplasia, Prostatic) OR (Prostatic Hypertrophy) OR (Adenoma, Prostatic) OR (Adenomas, Prostatic) OR (Prostatic Adenomas) OR (Prostatic Adenoma) OR (Prostatic Hyperplasia, Benign) OR (Prostatic Hypertrophy, Benign) OR (Benign Prostatic Hypertrophy) OR (Hypertrophy, Benign Prostatic) OR (“lower urinary tract symptoms”[Mesh]) OR (lower urinary tract symptom)] AND [(“Tadalafil”[Mesh]) OR (Pyrazino (1′,2′:1,6) pyrido (3,4-b)indole-1,4 -dione,6-(1,3-benzodioxol-5-yl)-2,3,6,7,12,12a-hexahydro-2-methyl-, (6R,12aR)-) OR (IC-351) OR (IC 351) OR (Cialis)]. Reference lists of relevant articles were also manually searched and the “related articles” function was used to identify additional articles in PubMed. The subsequent document filtration and data extraction were performed independently by the two authors (JW Cui, DH Cao), and conflicts on suitable articles were settled by discussion and consultation with two-third senior authors (Q Wei, J Wang).

### Study Selection

Trials included in our study met the following criteria according to the population, intervention, comparator, outcome, and study design (PICOS) approach: (P) male patients were diagnosed as LUTS/BPH; (I) taking medication: 12-week monotherapy with once daily 5 mg tadalafil; (C) treatment with an oral isodose placebo in the same way; (O) evaluating the following outcomes: changes in the total International Prostate Symptom Score (IPSS), IPSS storage (or irritative) subscore, IPSS voiding (or obstructive) subscore, IPSS quality of life (IPSS QoL), adverse events (AEs), and serious adverse events (SAEs); and (S) randomized controlled trials (RCTs). In addition, we excluded reviews, case reports, meeting abstracts, comments, letters to the editor, and animal research.

### Statistical Analysis

The outcome indicators were the QoL affected by LUTS/BPH scored using the IPSS, IPSS storage subscore, IPSS voiding subscore, BPH impact index (BII), IPSS QoL, the maximum flow rate (*Q*_max_), and the postvoid residual (PVR). Furthermore, safety-related findings assessed based on AEs and SAEs were additional indicators. Efficacy was evaluated by analyzing index changes from baseline to 12 weeks, whereas safety was assessed by comparing AE-related data. The analyses were mainly conducted using the Review Manager, version 5.4 (Cochrane Collaboration, Oxford, the UK). Specifically, heterogeneity was evaluated with Cochrane's Q-statistic test and the *I*^2^ tests. The random-effects model was used when heterogeneity was accepted at a *P*_*Q*_ < 0.10 or *I*^2^ > 50%, otherwise, the fixed-effects model was used. The risk of bias was independently assessed by two authors using the Jadad scale (≥3 scores represented high-quality studies), funnel plot, and *the Cochrane Collaboration's tool for assessing the risk of bias*, and conflicts were resolved by reaching a consensus through discussions. Sensitivity analysis was performed in the outcome indicators, respectively, by sequentially excluding each study. To find out more information about the ideal candidate for tadalafil monotherapy, several linear regressions were performed. Continuous variables reported as a mean and SD in the original articles were converted from standard error into SD for this study: SD = standard error ^*^(sample size)^1/2^. A 95% CI was chosen whether mean difference (MD) was used to express the continuous data or risk ratio (RR) was used for dichotomous data.

## Result

### Search Results and Quality Assessments

A total of 1,425 relevant articles were extracted from the databases through systematical and manual search strategies. Eventually, 13 articles (15–27) involving 9,525 participants were identified, of which 15 trials that met the eligibility criteria were included in this study. While the follow-up period was different in each study, we pooled data of the change from baseline to 12 weeks from the studies.

**Figure 1:**
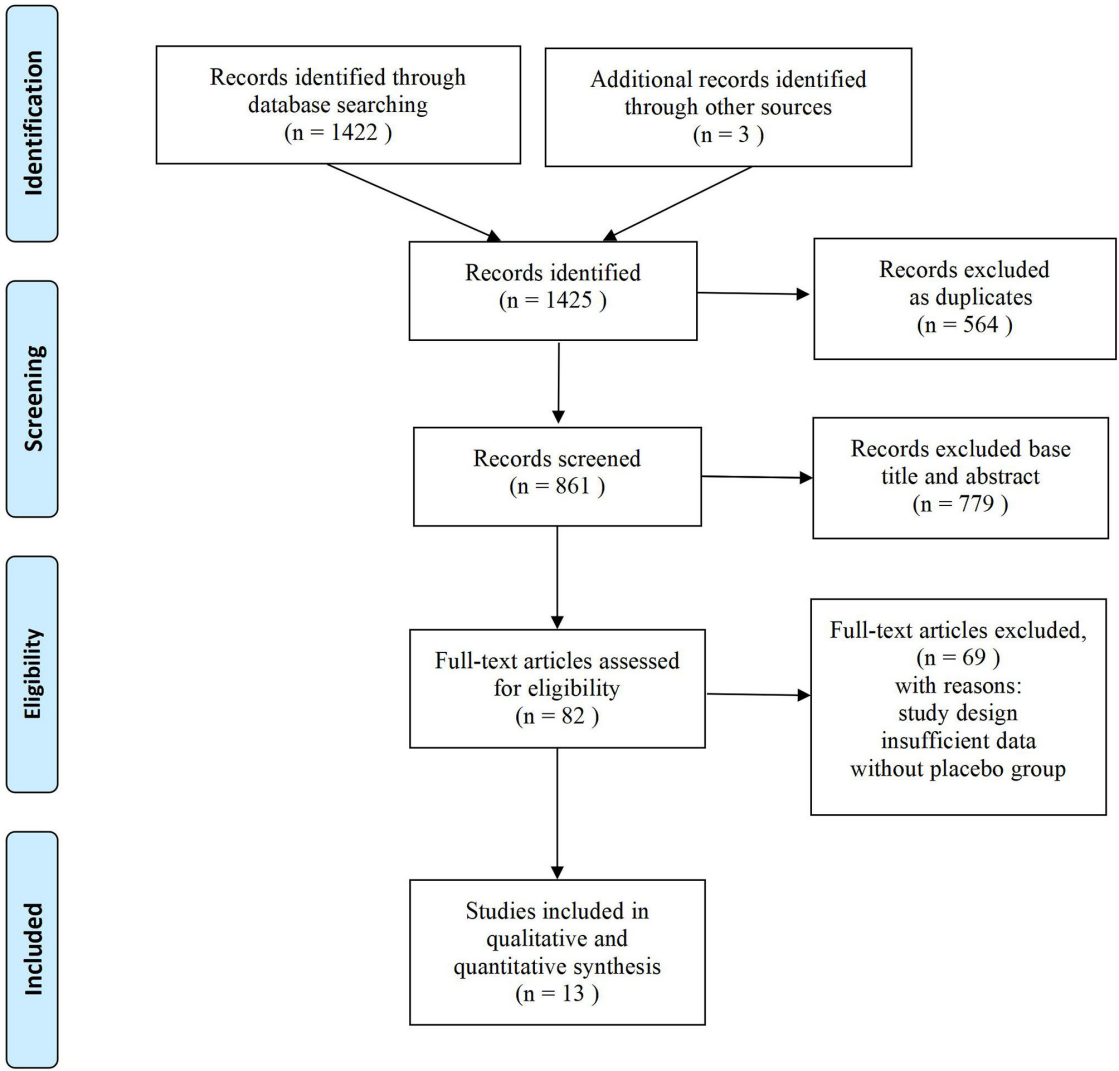
Flow diagram of literature searches.

**Figure 2:**
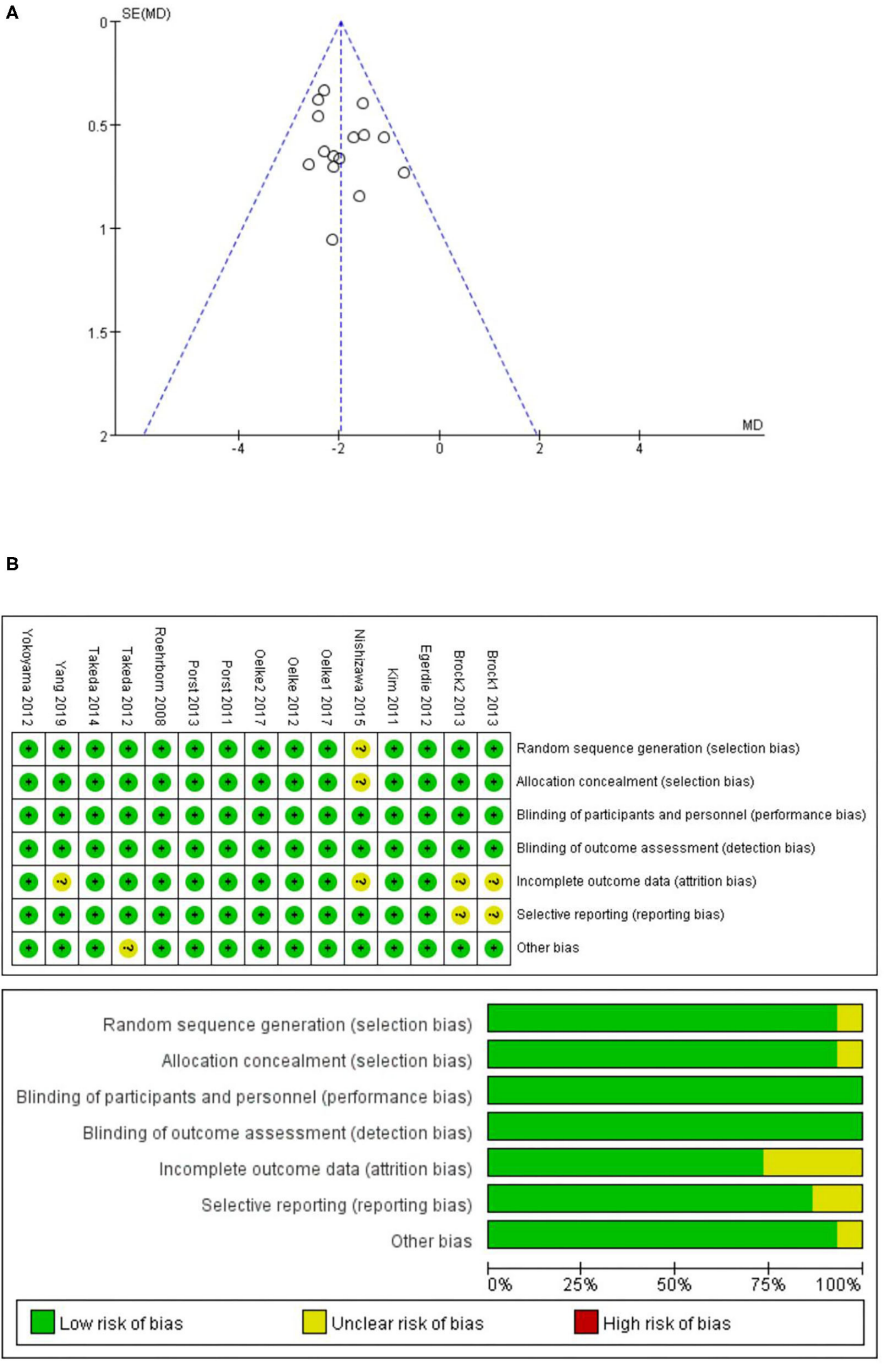
**(A)** Funnel plot of the studies represented in our meta-analysis; **(B)** risk of bias graph summary and risk of bias graph.

Figure 1 illustrates the literature search process used and Table 1 shows the mean baseline characteristics. The symmetrical funnel plot roughly revealed no selection biases or no small sample studies with poor methodological quality (Figure 2A). The risk of biases was presented further in the risk of bias graph summary and risk of bias graph, which illustrated that the probability of each bias in every study is quite small (Figure 2B). Apart from *Q*_max_, the rest of the indicators have reached the insubstantial heterogeneity standard (*P*_*Q*_ > 0.10 or *I*^2^ < 50% in forest plots) in Cochrane Reviewers' Handbook. In addition, no significant differences from the original analysis were found after sensitivity analysis.

**Table 1:** Participant demographic and baseline characteristics of the included studies.

	**Sample (*n*)**	**Age** **(years)**	**BMI** **(kg/m^**2**^)**	**PSA** **(ng/mL)**	**IPSS**	**IPSS** **storage**	**IPSS** **voiding**	**IPSS** **QoL**	**BII**	***Q*_**max**_** **(ml/s)**	**PVR** **(mL)**	**Jadad scale**
Roehrborn et al. (15)	212/ 211	61.95(46.10-85.60)/ 61.75 (45.59-80.62)	28.58(20.09-48.09)/ 28.57 (20.23-44.25)	1.79 (0.14-6.79)/ 1.73 (0.12-10.33)	17.30 ± 5.97/17.08 ± 6.36	NM	NM	NM	4.66 ± 2.98/ 4.94 ± 2.95	10.37 ± 3.86/ 10.31 ± 4.85	NM	5
Kim et al. (11)	51/ 51	61.2 ± 6.6/ 62.2 ± 6.8	24.7 ± 2.3/ 24.8 ± 2.2	1.0 ± 0.7/ 1.2 ± 1.0	17.1 ± 5.4/ 17.3 ± 5.0	NM	NM	NM	6.1 ± 2.9/ 6.2 ± 2.8	11.4 ± 3.2/ 10.5 ± 3.6	30.9 ± 33.8/ 34.4 ± 35.7	5
Porst et al. (23)	161/ 164	65.1 ± 8.4/ 64.6 ± 10.0	27.1 ± 3.8/ 28.4 ± 4.2	2.0 ± 1.8/ 2.2 ± 1.7	17.1 ± 6.1/ 16.6 ± 6.0	NM	NM	NM	5.1 ± 3.1/ 4.8 ± 3.2	11.6 ± 3.8/ 11.7 ± 4.4	44.9 ± 44.9/ 63.3 ± 59.9	5
Oelke et al. (19)	171/ 172	63.5(45.1–83.1)/ 63.7 (45.9–88.6)	27.1 (17.2–43.4)/ 28.1 (19.2–40.2)	2.1 ± 1.8/ 2.0 ± 1.7	17.2 ± 4.9/ 17.4 ± 6.0	6.8 ± 2.7/ 7.3 ± 3.2	10.5 ± 3.5/ 10.1 ± 4.1	NM	4.8 ± 2.8/ 5.0 ± 3.3	9.9 ± 3.6/ b10.5 ± 4.1	53.3 ± 50.4/ 50.2 ± 50.9	5
Egerdie et al. (18)	208/ 200	62.5 (45.7–82.0)/ 62.9 (45.4–83.2)	28.0 (19.8–43.2)/ 28.6 (17.7–51.5)	1.9 ± 1.8/ b 2.0 ± 1.8	18.5 ± 5.8/ 18.2 ± 5.3	NM	NM	NM	5.6 ± 3.1/ 6.0 ± 3.0	10.3 ± 3.5/ 10.1 ± 3.8	NM	5
Yokoyama et al. (21)	155/ 154	62.3 ± 8.0/ 63.7 ± 8.1	24.2 ± 2.8/ 24.3 ± 2.9	1.71 ± 1.14/ 1.74 ± 1.35	17.2 ± 6.0/ 16.8 ± 6.1	6.6 ± 2.6/ 6.7 ± 2.8	10.6 ± 4.4/ 10.0 ± 4.5	4.1 ± 1.2/ 3.9 ± 1.2	5.6 ± 3.1/ 5.2 ± 3.1	12.4 ± 4.8/ 12.3 ± 3.8	38.4 ± 51.2/ 41.9 ± 47.7	5
Takeda et al. (20)	140/ 140	67.4 (45.4-83.5)/ 67.4 (45.4-81.4)	23.5 ± 2.6/ 23.6 ± 2.9	NM	16.4 ± 5.9/ 16.5 ± 5.4	6.4 ± 2.7/ 6.2 ± 2.7	10.1 ± 4.3/ 10.3 ± 4.1	4.2 ± 1.1/ 4.2 ± 1.1	NM	11.5 ± 4.1/ 11.7 ± 4.4	32.2 ± 36.4/ 31.6 ± 42.7	5
Porst et al. (23)	521/ 505	62.9 ± 8.2/ 63.1 ± 8.2	27.7 ± 3.9/ 28.4 ± 4.4	1.9 ± 1.8/ 1.9 ± 1.7	17.6 ± 5.5/ 17.3 ± 5.8	7.5 ± 2.8/7.4 ± 2.9	10.1 ± 3.9/ 9.9 ± 3.9	3.6 ± 1.2/ 3.7 ± 1.3	5.2 ± 3.1/ 5.3 ± 3.2	10.6 ± 3.7/ 10.3 ± 3.7	NM	5
Brock et al. (22)	167/ 171	62.6 ± 8.3/ 61.2 ± 8.7	27.5 ± 3.7/ 28.3 ± 4.4	2.0 ± 1.6/ 1.9 ± 1.6	17.1 ± 6.3/ 16.8 ± 6.4	7.0 ± 3.2/ 7.2 ± 3.3	10.1 ± 4.3/ 9.6 ± 4.5	3.6 ± 1.4/ 3.6 ± 1.4	4.6 ± 2.8/ 4.9 ± 3.0	NM	NM	4
Brock et al. (22)	377/ 374	63.7 ± 8.3/ 64.1 ± 8.7	27.8 ± 4.1/ 28.4 ± 4.2	2.0 ± 1.7/ 1.9 ±1.6	17.3 ± 5.4/ 17.1 ± 6.0	7.4 ± 2.8/ 7.3 ± 3.0	9.9 ± 3.8/ 9.8 ± 4.1	3.6 ± 1.2/ 3.5 ± 1.2	4.9 ± 3.0/ 4.9 ± 3.2	NM	NM	4
Takeda et al. (24)	306/ 304	60.8 ± 7.7/ 60.9 ± 8.1	24.0 ± 3.0/ 24.1 ± 2.9	NM	18.7 ± 6.0/ 18.7 ± 5.2	7.1 ± 2.7/ 6.8 ± 2.7	11.7 ± 4.3/ 12.0 ± 3.7	4.3 ± 1.1/ 4.3 ± 1.1	NM	11.9 ± 4.5/ 11.9 ± 4.5	26.9 ± 37.7/ 32.7 ± 50.0	5
Nishizawa et al. (25)	601/ 98	62.6 ± 8.1/ 63.1± 8.2	23.9 ± 2.9/ 24.0± 2.9	1.9 ± 1.7/ 1.8 ± 1.6	17.8 ± 6.1/ 177 ± 5.6	NM	NM	4.2 ± 1.1/ 4.2 ± 1.1	NM	12.0 ± 4.6/ 11.9 ± 4.3	NM	3
Oelke et al. (24)	1662/ 1350	58.6 ± 9.1/ 60.0 ± 8.4	27.4 ± 4.3/ 27.5 ± 4.6	NM	17.4 ± 5.9/ 17.2 ± 5.9	7.1 ± 2.9/ 7.1 ± 2.9	10.3 ± 4.1/ 10.1 ± 4.1	3.8 ± 1.3/ 3.8 ± 1.3	NM	NM	NM	5
Oelke et al. (24)	154/ 143	77.2 ± 2.6/ 77.6 ± 3.0	26.1 ± 4.2/ 26.5 ± 4.1	NM	17.1 ± 5.1/ 17.0 ± 5.8	7.6 ± 2.9/ 7.5 ± 2.8	9.6 ± 3.8/ 9.6 ± 4.3	3.7 ± 1.3/ 3.7 ± 1.2	NM	NM	NM	5
Yang et al. (27)	51/ 51	60.8 ± 7.3/ 62.6 ± 6.5	24.6 ± 3.8/ 24.3 ± 2.2	1.2 ± 1.0/ 1.2 ± 1.0	14.7 ± 6.0/ 14.4 ± 6.7	5.8 ± 2.7/ 5.5 ± 2.9	8.9 ± 4.7/ 8.9 ± 4.8	NM	NM	16.1 ± 7.8/ 18.6 ± 9.7	21.3 ± 19.2/ 20.8 ± 36.1	4

*Continuous variables were reported as mean ± SD or as median (range)*.

*T/P, the tadalafil group/the placebo group; BMI, body mass index; PSA, prostate specific antigen; IPSS, International Prostate System Score; QoL, quality of life; BPH, benign prostatic hyperplasia; BII, BPH impact index; Q_max_, maximum flow rate; PVR, postvoid residual; NM, not mention*.

### Efficacy

#### Total IPSS

**Figure 3:**
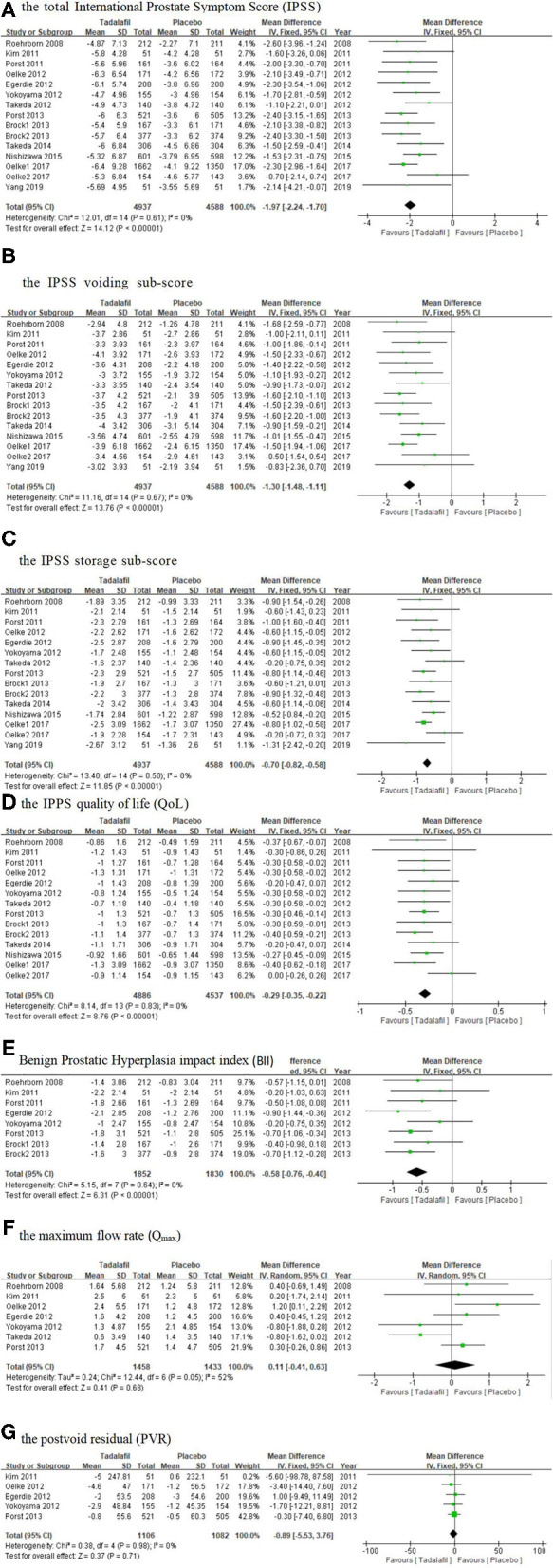
Forest plot of **(A)** the total IPSS; **(B)** the IPSS voiding subscore; **(C)** the IPSS storage subscore; **(D)** the IPSS QoL; **(E)** BII; **(F)** *Q*_max_; and **(G)** PVR. IPSS, International Prostate Symptom Score; PVR, postvoid residual; QoL, quality of life.

The total IPSS, IPSS voiding subscore, and IPSS storage subscore were reported in all the trails we selected. The data pooled from 15 studies (15–27) including 9,525 participants (4,937 and 4,588 in the tadalafil and placebo groups, respectively), and provided the necessary information for this study. The subsequent analysis result revealed a significantly larger total IPSS change from the baseline to 12 weeks in the tadalafil group (MD: −1.97 95% CI: −2.24 to −1.70, *P* < 0.00001, Figure 3A) than in the placebo group.

#### IPSS Voiding Subscore and IPSS Storage Subscore

A total of 15 studies (15–27) included in the analysis recorded the change in the IPSS voiding and storage subscores from baseline to 12 weeks. The analysis of the combined data showed that tadalafil significantly reduced the IPSS voiding and storage subscores more than the placebo did, and the MD of the change in the two subscores was −1.30 (95% CI: −1.48 to −1.11, *P* < 0.00001, Figure 3B) and −0.70 (95% CI: −0.82 to −0.58, *P* < 0.00001, Figure 3C), respectively.

#### The IPSS Quality of Life

A total of 14 studies (15–26) including 9,423 participants (4,886 and 4,537 in the tadalafil and placebo groups, respectively) investigated the IPSS QoL improvement data. The fixed-effects estimate of the MD was −0.29 (95% CI: −0.35 to −0.22, *P* < 0.00001, Figure 3D).

#### BPH impact Index

Eight studies (15–18, 21–23) including 3,682 participants (1,852 and 1,830 in the tadalafil and placebo groups, respectively) compared the effect of the treatments on the BII. Overall, the pooled MD was −0.58 (95% CI: −0.76 to −0.40, *P* < 0.00001, Figure 3E) in the fixed-effect model, which indicated the BII was significantly lower in patients administered tadalafil therapy than in those who received the placebo.

**Figure 4:**
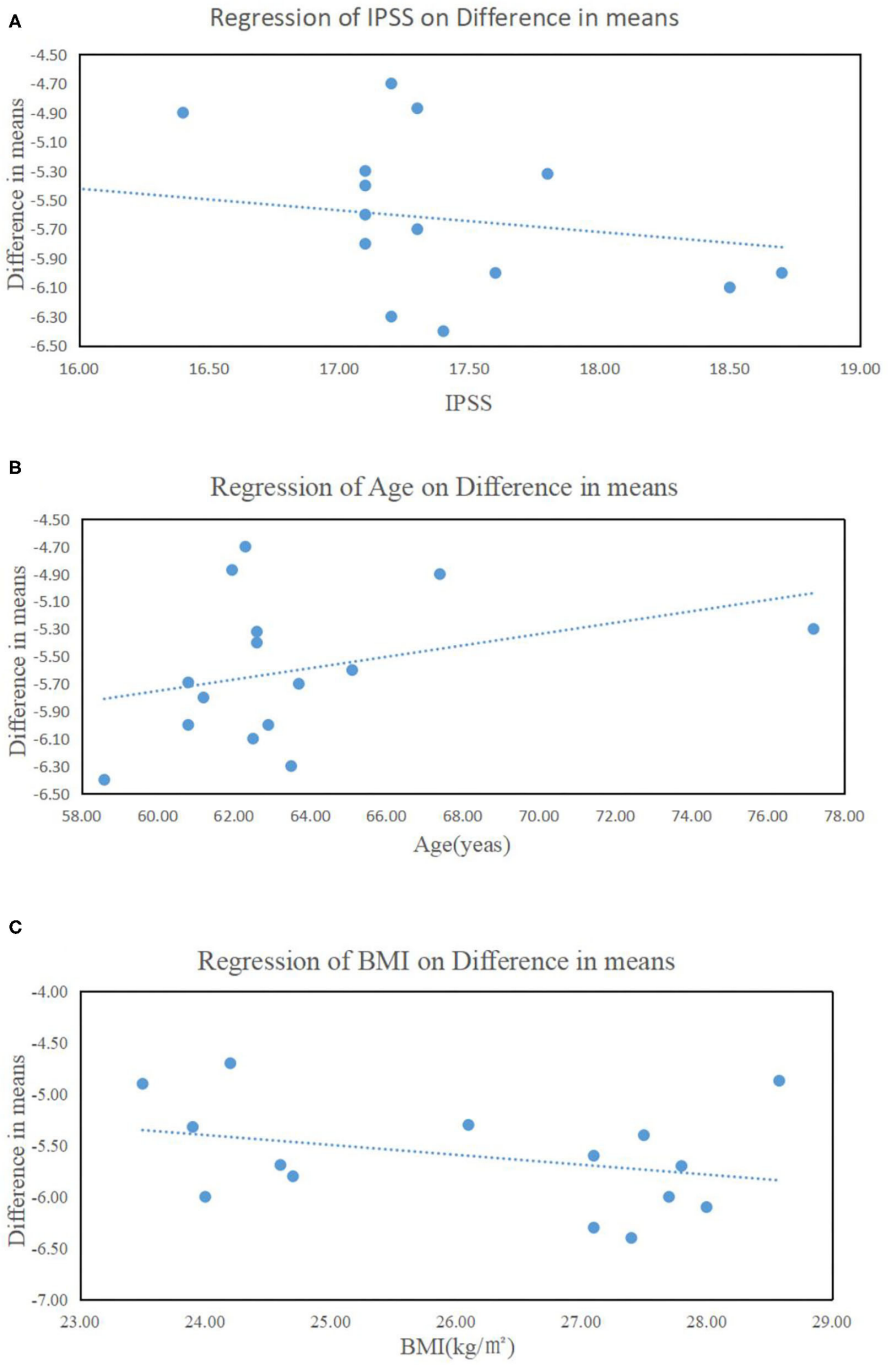
Influence of **(A)** the baseline of the total International Prostate Symptom Score (IPSS), **(B)** age, and **(C)** body mass index on IPSS improvement.

These results suggested that tadalafil oral monotherapy (5 mg once daily for 12 weeks) significantly improved LUTS/BPH in the treated patients. Interestingly, the better improvement in IPSS seemed to be in connection with the younger age, the high BMI, and the high baseline IPSS (Figure 4).

#### The Maximum Flow Rate (Q_max_)

The mean change in the *Q*_max_ was calculated from pooled data from seven studies (15, 16, 18–21, 23) including 2,891 participants (1,458 and 1,433 in the tadalafil and placebo groups, respectively). The results showed an insignificant difference between tadalafil and the placebo, with an MD of 0.11 (95% CI: −0.41 to 0.63, *P* = 0.68, Figure 3F).

#### Postvoid residual

Five studies (16, 18, 19, 21, 23) including 2,188 participants (1,106 and 1,082 in the tadalafil and placebo groups, respectively), provided information about the change in the PVR from baseline to 12 weeks. The results indicated that the decrease in PVR was similar between these two groups, with an MD of −0.89 (95% CI: −5.53 to 3.76, *P* = 0.71, Figure 3G).

#### Safety

In our study, the most common AEs were headache (3.59%), nasopharyngitis (3.21%), dyspepsia (2.92%), and back pain (2.29%). The results of meta-analyses showed that a higher incidence of headache, dyspepsia, and back pain was associated with tadalafil monotherapy (Table 2).

**Table 2:** Most common reported treatment-related adverse events.

**Study (T/P)**	**Sample (*n*)**	**Headache**	**Nasopharyngitis**	**Dyspepsia**	**Back pain**	**Dizziness**	**Myalgia**	**Extremity pain**
Roehrborn et al. (15)	212/211	6/6	4/2	10/0	2/1	NM	3/0	5/0
Kim et al. (11)	51/51	1/1	1/0	0/0	NM	NM	3/0	NM
Porst et al. (23)	161/164	6/1	NM	NM	5/4	4/3	NM	NM
Oelke et al. (19)	171/172	5/2	5/8	4/0	4/1	NM	NM	NM
Egerdie et al. (18)	208/200	12/6	5/4	NM	6/3	NM	NM	NM
Yokoyama et al. (21)	155/154	3/1	2/4	NM	4/1	0/0	6/0	NM
Takeda et al. (20)	140/140	3/3	14/18	4/0	2/2	NM	NM	NM
Porst et al. (23)	521/505	20/13	NM	11/1	13/6	NM	NM	NM
Brock et al. (22)	167/171	7/2	6/4	6/0	5/3	1/2	5/1	2/0
Brock et al. (22)	377/374	10/7	6/9	9/1	7/3	7/2	2/1	8/0
Takeda et al. (24)	306/304	9/6	13/10	12/2	NM	NM	NM	NM
Nishizawa et al. (25)	601/598	15/10	29/32	18/2	NM	3/2	NM	NM
Oelke et al. (19)	1662/1350	75/35	43/40	52/5	40/15	11/10	NM	21/0
Oelke et al. (19)	154/143	3/1	7/8	2/0	2/2	5/1	NM	2/0
Yang et al. (21)	51/51	2/1	NM	1/0	NM	NM	2/0	NM
Total								
Trials	15	15	12	12	11	7	6	5
Tadalafil group	4,937	177/4,937 (3.59%)	135/4,204 (3.21%)	129/4,413 (2.92%)	90/,3928 (2.29%)	31/3,287 (0.94%)	21/1,013 (2.07%)	38/2,572 (1.48%)
Placebo group	4588	95/4,588 (2.07%)	139/3,868 (3.59%)	11/4,070 (0.27%)	41/3,584 (1.14%)	20/2,962 (0.68%)	2/1,012 (0.20%)	0/2249(0%)
Heterogeneity								
p-value		0.99	0.95	1.00	0.99	0.51	0.95	0.84
I^2^		0	0	0	0	0	0	0
RR [95% CI]		1.70 [1.33, 2.18]	0.91 [0.72, 1.14]	8.67 [5.01, 15.02]	2.00 [1.39, 2.89]	1.41 [0.81, 2.46]	5.76 [2.00, 16.59]	14.87 [4.08, 54.18]
Z		4.24	0.83	7.71	3.70	1.21	3.25	4.09
*p*-value		<0.0001	0.40	<0.00001	0.0002	0.23	0.001	<0.0001

*T/P, The tadalafil group/the placebo group; NM, not mention*.

**Figure 5:**
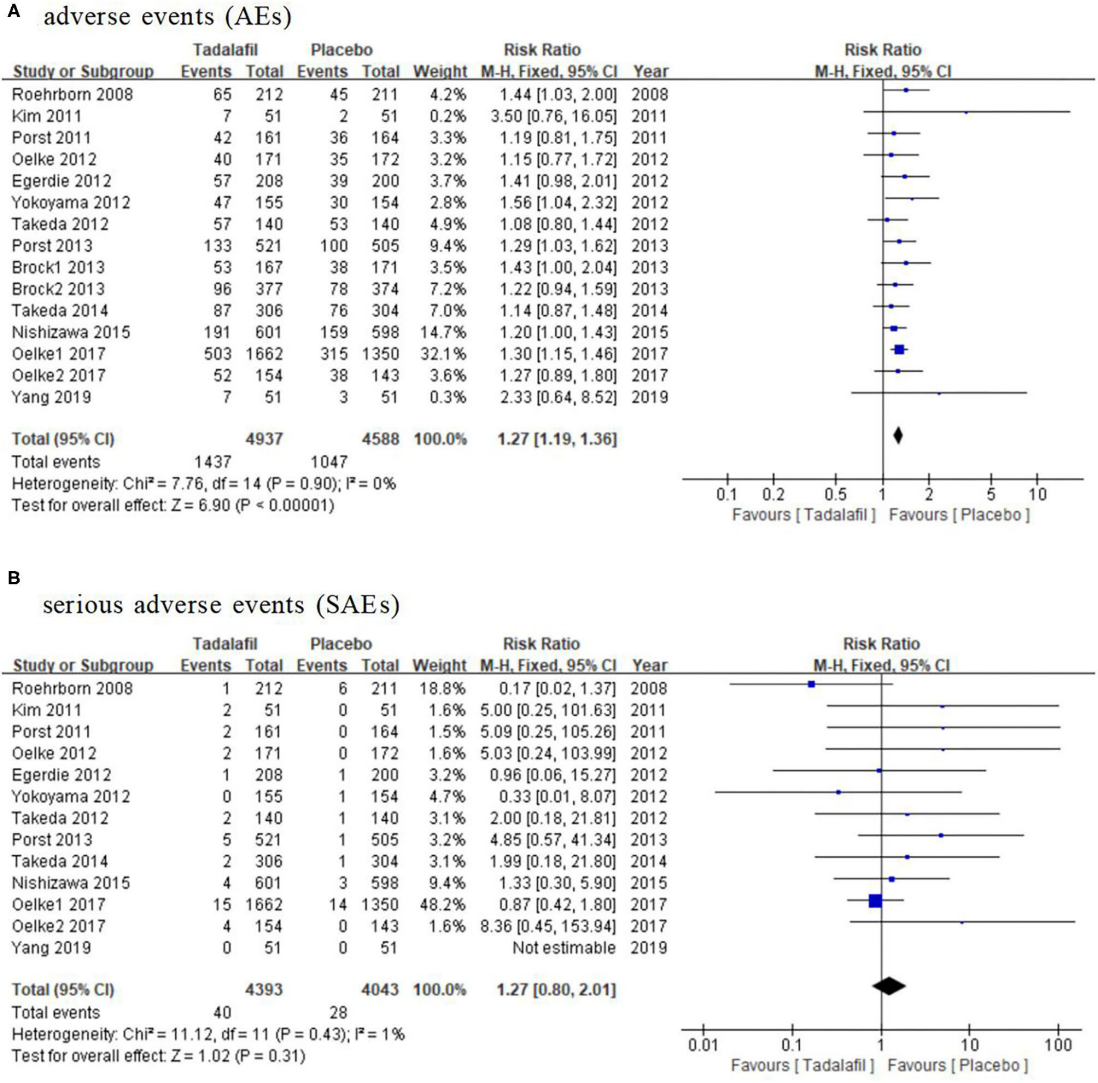
Forest plot of **(A)** AE and **(B)** SAE. AE, adverse event; SAE, serious adverse event.

The AEs and SAEs that occurred in the tadalafil and placebo groups were compared in this review. All the studies included (15–27) in the analysis reported the incidence of AEs, which showed a significant difference between the tadalafil and placebo groups with a RR of 1.27 (95% CI: 1.19–1.36, *P* < 0.00001, Figure 5A). However, the comparison of incidences of SAE did not show any significant difference between the two groups (RR: 1.27, 95% CI: 0.80–2.01, *P* = 0.31, Figure 5B) in the 13 studies including 8,436 participants (4,393 and 4,043 in the tadalafil and placebo groups, respectively).

## Discussion

This systematic review and meta-analysis have demonstrated comprehensively the therapeutic effect and clinical safety of 12-week monotherapy with once daily 5 mg tadalafil for LUTS/BPH. In our study, the total IPSS improvement was clinically meaningful after a tadalafil monotherapy, which significantly improved the IPSS voiding and storage subscores, IPSS QoL, and BII, whereas no statistically significant difference was found in terms of *Q*_max_ and PVR. In addition, the result shows that tadalafil and placebo were similar in the matter of the incidence of SAEs, although the difference in AEs was statistically significant.

Medicinal management has become the standard of therapy in patients diagnosed with moderate to severe LUTS/BPH, whereas surgery is performed in those who fail to respond to medication. Tamsulosin is regarded as an effective treatment for LUTS/BPH because it selectively blocks α_1_ receptors to induce smooth muscle relaxation in the bladder, neck, and prostate (28). Furthermore, tamsulosin 0.4 mg administered once daily is used in Western countries and is the safest AB in combination with PDE5Is (12). As a recommended medicine, tamsulosin could improve the symptom score by 4–6 points in the European Association of Urology guidelines. Likewise, the total IPSS was reduced by an average of 4.7–6.4 points with tadalafil monotherapy in the studies we included. Coincidentally, the study by Oelke (19), comparing with tamsulosin, monotherapy with tadalafil seemed to be a similar therapeutic effect in improving moderate to severe LUTS/BPH. All these make it necessary to test whether tadalafil is effective in LUTS/BPH with more sufficient evidence.

Tadalafil, the only PDE5I used for the treatment of BPH, was initially prescribed for the treatment of ED in 2003 but later proved to be effective in LUTS/BPH. Consequently, this agent was approved by the US Food and Drug Administration (FDA) in 2011 for the treatment of symptoms of BPH especially when accompanied by ED (29). A dose-finding study was conducted with a sample size of 1,058 patients experiencing LUTS/BPH who were randomly assigned to receive tadalafil (2.5, 5, 10, or 20 mg) or a placebo once daily. The results showed that tadalafil administered at all the tested doses significantly improved the symptoms more than the placebo did, and tadalafil 5 mg daily showed a more positive risk-benefit profile (15). In fact, an oral dose of 5 mg per day is the standard for tadalafil (12). Dong et al. (30) performed a meta-analysis of studies reporting the use of monotherapy for LUTS in 2012, whereas Wang et al. (31) conducted a similar study in 2016. However, the tadalafil dose used in the included trials in the article by Dong et al. (30) was not unified in the analysis of the comprehensive effect of four different doses, and the number of studies included was small in the latter passage (30, 31). Furthermore, three related literature reports (25–27) (four RCTs) have been published over the last 5 years. Therefore, the latest and largest pooled analysis and systematic review are urgently required to evaluate the therapeutic effect and clinical safety of tadalafil with a unified dose (5 mg once daily) and a uniform endpoint (12 weeks) in patients with LUTS/BPH.

The reduced indexes, including the total IPSS, IPSS voiding and storage subscores, IPSS QoL, and BII, showed the relief of symptoms of BPH and the improvement of QoL. As we all know, IPSS QoL indicates the view of the patient if always accompanied by current LUTS/BPH in a future life, among which are six options: from 0 (pleasurable) to 3 (acceptable), to 6 points (terrible), in turn. Therefore, although the score of QoL was only reduced by about one point, it meant a qualitative leap in the satisfaction of patients with the improvement of urination symptoms and reflected the need of the patient for treatment. Storage symptoms, especially the relief in nocturia, made a greater contribution to the improvement in QoL (32). These statistically significant results illustrated a great therapeutic effect of tadalafil in improving LUTS, but the specific mechanisms of action remain unclear. The PDE5 enzyme is expressed in the bladder, urethra, corpora cavernosa, prostate, ureter, and kidney (33). Matsukawa et al. (34) conducted a urodynamic-based study that demonstrated that tadalafil significantly improved bladder outlet obstruction and bladder storage function. In addition, *in vitro* studies showed that a PDE5I relaxed the smooth muscle of the bladder, neck, and prostate, and decreased detrusor muscle overactivity (35). The relaxant effect of PDE5Is on the bladder neck and urethra mediated by the NO-cGMP signaling pathway might result in symptom relief in patients with LUTS/BPH. Other related mechanisms include decreased activation of the RhoA/Rho kinase pathway, improvement of oxygenation, regulation of proliferation and transdifferentiation, modulation of the afferent nerves of the bladder and the prostate along with autonomic nervous system overactivity, and anti-inflammatory effects (7, 36). These mechanisms identified by our results might mediate the effective alleviation of the symptoms of BPH by this agent.

The corpus cavernosum, bladder, and prostate have similar pathophysiological pathways, and, therefore, LUTS/BPH and ED are often comorbid in the older population and have a higher incidence in older men (37). More importantly, LUTS is considered a risk factor for ED (38). Although many PDE5Is have similar mechanisms of action, tadalafil is the only PDE5I approved for the treatment of BPH because of its safety, tolerability, and longer duration of action (7). Nevertheless, a study of more than 1,000 men conducted by Broderick et al. (39) reported that tadalafil exhibited a similar efficacy in improving LUTS/BPH in men with or without comorbid ED. Moreover, Dong et al. (30) performed a subgroup analysis and reported that tadalafil showed a more significant improvement in BPH symptoms in men without ED than in those with ED. The effect of tadalafil on LUTS does not appear to mainly involve its mechanism of action on ED. However, the inhibitory effects of tamsulosin on neurogenic contractions have been reported to be enhanced by tadalafil in the human bladder, neck, and prostate. Those findings along with the study showing that cotreatment with ABs and tadalafil produces an additional relaxation effect on the corpus cavernosum *in vitro* (40, 41), suggests there might exist synergistic effects among tadalafil and tamsulosin accounting for the better effect in patients who are suffering from LUTS/BPH and ED. Thus, when an ABs or tadalafil alone does not show sufficient efficacy, cotherapy with both agents may be considered.

No statistically significant difference was found in *Q*_max_ and PVR in this study, which is consistent with the finding by Liu et al. (42). However, trials studying cotreatment with ABs and PDE5Is demonstrated that it increased the urinary flow rate (43, 44). The relaxant effect of PDE5Is on the bladder neck and prostate, which should have increased the urinary flow, might be counteracted by the concomitant relaxation of the detrusor muscle. Nevertheless, cotherapy may exert a synergistically positive effect on the detrusor muscle, thereby improving the urinary flow rate (12). More studies are required to elucidate the mechanism.

Although the difference in AEs was statistically significant, the side effects were mild and sustainable, such as headache, dyspepsia, and back pain. Furthermore, the difference in the incidence of SAEs was not significant. Interestingly, one patient reported an SAE in the tadalafil group whereas six participants reported in the placebo group of the study by Roehrborn (15) reported SAEs, suggesting that some AEs might be caused by psychological effects. Moreover, Wada et al. (45) demonstrated that the main reason for withdrawal of tadalafil was insufficient efficacy, followed by AEs and improvement of symptoms (45). In addition, calculated finally to be 2.5% on average, the incidence of discontinuation was low. Therefore, tadalafil 5 mg daily had no obvious adverse effects and is highly effective in improving LUTS/BPH.

Previous trials have reported that a higher baseline IPSS score correlates with a better therapeutic effect of ABs (46), which is consistent with our present observation (Figure 4A). Moreover, the improvement in IPSS appeared to be related to age and obesity (Figures 4B,C). These results suggested that younger, more obese men with more severe urinary symptoms may be the ideal candidates for tadalafil monotherapy. This could be explained by the changes in metabolism and hormonal levels that accompany aging and obesity. Testosterone levels decrease with increasing age and the hormonal dependency of PDE5 expression has been found in the animal models (47). Additional research is needed to further verify these results and explore the underlying mechanisms of action.

There are some limitations to our study that should be recognized. We were restricted to studies comparing tadalafil with placebo, rather than other pharmacological treatments recommended in guidelines to manage LUTS. Nonetheless, we focus on whether tadalafil is effective and safe in treating LUTS/BPH, rather than assessing which is better after comparing tadalafil and other drugs. In addition, relatively few experiments reported *Q*_max_ and PVR, and those that did were conducted before 2013, which may have caused the lack of statistical significance observed. More large-scale, well-designed, and long-term prospective clinical studies using comprehensive indicators should be conducted to further confirm the effectiveness and safety of tadalafil monotherapy in improving LUTS/BPH vs. active control.

Despite the several limitations to this study, we have reported the latest and most comprehensive evidence-based medical proof to demonstrate that 5 mg once daily tadalafil was effective and safe for the treatment of LUTS/BPH. All the studies in our analysis were RCTs and the follow-up deadline of 3 months was long enough, and the heterogeneity of our forest plots was also calculated to be low, except for the *Q*_max_ (*I*^2^ = 52%). These findings indicate the reliability of the curative effect of tadalafil. As the population ages, more and more aging men are being troubled with LUTS/BPH together with ED. As an effective treatment for ED, tadalafil, shown in our study, has also a therapeutic effect in relieving LUTS/BPH. So, tadalafil is a better choice for men suffering from both LUTS/BPH and ED. What is more, due to its safety and tolerable AEs, tadalafil may provide another option for those who are intolerant to the side effects of ABs or 5ARIs. This study could provide a more meaningful reference for clinicians and researchers, and provide more concrete evidence for future guidelines for the treatment of BPH.

## Conclusion

Treatment with 5 mg tadalafil once daily effectively improved total IPSS, IPSS voiding subscore, IPSS storage subscore, IPSS QoL, and BII. Moreover, monotherapy with tadalafil appeared to be well tolerated and safe because no significant difference was detected in SAEs between the tadalafil- and placebo-treated patients. Finally, 5 mg tadalafil administered once daily could be a therapeutic option for patients with LUTS/BPH.

## Data Availability Statement

The original contributions presented in the study are included in the article/supplementary material, further inquiries can be directed to the corresponding authors.

## Author Contributions

Conception and design, and administrative support by JW and QW. Provision of study materials or patients were administered by JC and DC. Collection and assembly of data were administered by JC, DC, YB, WW, and YX. Data analysis and interpretation were administered by JC, DC, and YB. Manuscript writing and final approval of manuscript were done by all the authors.

## Funding

This work was funded by the National Natural Science Foundation of China (Grant no. 82000721 and no. 81770756); the Post-Doctor Research Project, West China Hospital, Sichuan University (Grant no. 2019HXBH089); and the Health Commission of Sichuan Province (Grant no. 20PJ036).

## Conflict of Interest

The authors declare that the research was conducted in the absence of any commercial or financial relationships that could be construed as a potential conflict of interest.

## Publisher's Note

All claims expressed in this article are solely those of the authors and do not necessarily represent those of their affiliated organizations, or those of the publisher, the editors and the reviewers. Any product that may be evaluated in this article, or claim that may be made by its manufacturer, is not guaranteed or endorsed by the publisher.
